# Polish pseudo-words list: dataset of 3023 stimuli with competent judges’ ratings

**DOI:** 10.3389/fpsyg.2015.01395

**Published:** 2015-09-15

**Authors:** Kamil K. Imbir, Tomasz Spustek, Jarosław Żygierewicz

**Affiliations:** ^1^Faculty of Psychology, University of WarsawWarsaw, Poland; ^2^The Maria Grzegorzewska UniversityWarsaw, Poland; ^3^Faculty of Physics, University of WarsawWarsaw, Poland

**Keywords:** pseudo-words, lexical stimuli dataset, polish language, lexical decision task, lexical processing

## Abstract

Pseudo-words are stimuli, which are useful in research concerning lexical processing. As in the case of existing words, they are language dependent; thus, they should be generated for each language separately. The Polish Pseudo-words List (PPwL) is a dataset presenting a set of 3023 stimuli (words of 4–13 letters long). They were generated using an algorithm substituting random letters in existing words with respect to the frequency of letters in certain positions. We put out the raw set for a competent judges’ assessment and included the responses in the dataset. PPwL allows the choice of suitable control stimuli for experiments concerning lexical processing.

## Introduction

Studies in psychology concerning language processing in so-called lexical decision tasks (c.f. Meyer and Schvaneveldt, 1971) require both words of well-known properties (c.f. Imbir, 2014) and pseudo-word stimuli (e.g., Simos et al., 2002; Keuleers and Brysbaert, 2010) following some orthographical and structural rules (c.f. judging procedure). They should especially respect the phonotactic restrictions of a certain language; thus, each needs their own pseudo-word stimuli, respecting language’s specificity. Pseudo-word stimuli have no meaning in the lexicon, but it is possible that such stimuli could potentially be a part of the language. Using proper stimuli is especially important when processing differences are measured in EEG paradigms (c.f. Kanske and Kotz, 2007; Barber et al., 2013; Palazova et al., 2013; Imbir et al., submitted) that are sensitive to subtle differences in stimuli classes. Pseudo-words are more complex forms of stimuli than logatomes or non-sense syllables both of which are composed of single syllables. For that reason, to create them, we may use existing syllables as well as artificial (yet pronounceable) ones. Although in the literature the machine pseudo-words generation method exists (c.f. Keuleers and Brysbaert, 2010), at the moment of beginning of our project “Wuggy” generator was not customized to Polish language. For that reason we decided to generate pseudo-words in a different random fashion (but respecting letters probability of occurrence on certain position in certain neighborhood) and then put all of them into judging procedure.

The aim to create the presented dataset was to provide a set of stimuli (varying with degree of fulfillment of the criteria of ideal pseudo-word) for experimental samples in Polish language. To make use of pseudo-word stimuli easier for other researchers, we decided to share our dataset of 3023 pseudo-words. We hope that this will stimulate research on lexical processing in studies using the Polish language. This could lead to a better understanding of word processing in diverse languages.

## Materials and Methods

### Pseudo-Word Generation

The generation procedure engaged two steps. At first, we chose 540 random nouns from a normative database of 4905 Polish words (Imbir, submitted). We wanted them to cover words of different lengths (number of letters ranged from 4 to 13). Then, for each noun chosen, six machine-generated pseudo-words were constructed by substituting randomly selected letters for other letters. These other letters retained their type – vowel or consonant – and had to be one of the three most probable to occur after the preceding and before the successive letter. In fact most probable letter (or letters – the algorithm was random so the same letter position could have been chosen twice or more and thus generate two different pseudo-words) for certain, randomly chosen position was placed instead of original letter. The original letter at chosen position was excluded, so if that letter was most probable to occur at certain position, algorithm replaced it by second most probable. Also if generated stimulus was the same as previously generated (or other existing word included in 4905 words list) algorithm searched for another pseudo-word in order to replace this one. As reference point for probability of occurrence we used whole 4905 word list (Imbir, submitted) representing large number of words from Polish language. The rationale for this choice was expectation that generated pseudo-words should match as much as possible to available lexical stimuli (whole list). Unfortunately, this procedure does not guarantee that pseudo-words respect the phonotactic restrictions of the language, thus further judge competent engagement was crucial. For words of 4–6 letters long, one letter was substituted; for words of 7–9 letters, two letters were substituted, and for words 10–13 letters long, three letters were substituted. In this way, we obtained a list of 3240 pseudo-words.

### Judging Procedure

The third step was to evaluate our list in terms of subjective fulfillment of criteria for pseudo-word stimuli by using competent judges. Pseudo-words were defined as verbal stimuli that (1) are constructed from existing or potential syllables, (2) are possible to read fluently, (3) comply with Polish spelling rules, (4) do not occur in the real language, and (5) do not associate easily with other existing words in the language. We asked five native Polish language speakers (women), who were students of social science and humanities (including departments for language and literature) to evaluate the whole set of 3240 stimuli and remove those items from the list that did not conform to all of the criteria. After this validation, we inspected the judges’ congruency concerning individual pseudo-word stimuli. The advantage of presented methodology is that we asked judges to exclude pseudo-words that can be easily associated with existing words or hard to read and present instant list. Judging is still often needed in case of other pseudo-words stimuli generation procedures.

Eight hundred seventy pseudo-words were positively verified by all five judges and received a congruency index of 1. Next, 988 stimuli were chosen by four of the five judges at the same time (congruency index = 0.8). A total of 537 pseudo-words were indicated by three of the five judges as good stimuli with a congruency index of 0.6. Two judges agreed in the case of 341stimuli (congruency index = 0.4); for 287 stimuli, only one judge indicated that they were good pseudo-words, while the other four crossed these stimuli out (congruency index = 0.2). Two hundred seventeen (6,7% of initial number) stimuli were excluded by all of competent judges.

## Dataset Description

The Polish Pseudo-words List (PPwL) dataset is deposited at http://figshare.com/s/1089daa40de311e589a806ec4b8d1f61 and consists of a single xlsx spreadsheet. Pseudo-words are listed in the first column. In the next two columns, one can find the agreement ratio for every single pseudo-word as well as the number of judges indicating that the certain stimuli is a good example of a pseudo-word (max = 5). In the last column, stimulus length is presented as the number of letters in the string. We may assume that the number of 870 pseudo-words with maximum judges congruency represent stimuli of very good quality adhering to five criteria listed above.

## Conflict of Interest Statement

The authors declare that the research was conducted in the absence of any commercial or financial relationships that could be construed as a potential conflict of interest.
